# Dimethylamine Borane Neurotoxicity

**DOI:** 10.1289/ehp.114-a274a

**Published:** 2006-05

**Authors:** Chin-Chang Huang, Hung-Chou Kuo

**Affiliations:** Chang Gung Memorial Hospital, Taipei, Taiwan, E-mail: cch0537@adm.cgmh.org.tw

In a recent article, Tsan et al. (2005) reported the first study of the clinical toxicity of dimethylamine borane (DMAB) poisoning, particularly the neurologic manifestations in the central nervous system and peripheral nervous system. They decribed a prominent cerebellar dysfunction including impairments in performing tandem gait, finger-to-nose, and heel-to-knee tests and slurred speech in one patient who had the most severe neurologic symptoms 3 days after DMAB intoxication. We had the opportunity to evaluate the same patient 1 month after DMAB intoxication (Kuo et al., in press). Neurologic examinations revealed no cerebellar signs. Although Tsan et al. (2005) reported that brain magnetic resonance images (MRI) showed a symmetric increase in signal intensity at the bilateral cerebellar periventricular areas on 9 February 2004, the follow-up brain MRI study in our hospital 6 weeks after intoxication showed a nearly normal result. However, Tsan et al. (2005) interpreted that the rapid recovery of brain MRI changes may be due to a transient demyelination, axonal degeneration, or neuronal damages. This phenomenon is best interpreted as a transient brain edema. In addition to the transient edema, the other possibility is that the high signal intensity in cerebellar dentate nuclei is caused by an artifact. Therefore, a series section of the brain MRI images is required.

Six weeks after DMAB exposure, we found that the patient was still confused about time (Kuo et al., in press). Fourteen weeks after exposure, a neuropsychological study revealed that he had a cognitive impairment in learning ability of verbal and nonverbal learning memory; attention functions including focus, tracking, and divided attention; working memory; and semantic category retrieval. The data suggested that a prolonged toxic effect of the DMAB on the central nervous system still persisted in the attention, personality, and memory function.

The patient developed a progressive distal numbness and limb weakness 5 days after DMAB intoxication, and neurologic examinations showed generalized hyporeflexia; decreased sensation in pin-prick, temperature, touch, vibration, and position sensations; and weakness in the distal limbs, particularly in both feet (Kuo et al., in press). The data were further supported by sural nerve biopsy and cutaneous nerve biopsy studies (Kuo et al. in press). Our serial nerve conduction studies also confirmed axonal polyneuropathy, particularly in the motor nerves. However, Tsan et al. (2005) stated that the polyneuropathy with axonal polyneuropathy was verified by serial electroencephalogram and nerve conduction velocity studies.

Our time course of DMAB-induced polyneuropathy indicated an acute axonal polyneuropathy (Kuo et al., in press). However, Tsan et al. (2005) misdiagnosed the patient as having a delayed polyneuropathy, which may be wrong, because the time course was different from the so-called “delayed polyneuropathy” induced by organophosphate intoxication (Senanyake and Karalliedde 1987).
